# Characterization of Glycosylation-Specific Systemic and Mucosal IgA Antibody Responses to *Escherichia coli* Mucinase YghJ (SslE)

**DOI:** 10.3389/fimmu.2021.760135

**Published:** 2021-12-17

**Authors:** Saman Riaz, Hans Steinsland, Mette Thorsing, Ann Z. Andersen, Anders Boysen, Kurt Hanevik

**Affiliations:** ^1^ Department of Clinical Science, University of Bergen, Bergen, Norway; ^2^ Centre for International Health, Department of Global Public Health and Primary Care, University of Bergen, Bergen, Norway; ^3^ Centre for Intervention Science in Maternal and Child Health (CISMAC), Centre for International Health, Department of Global Public Health and Primary Care, University of Bergen, Bergen, Norway; ^4^ Department of Biomedicine, University of Bergen, Bergen, Norway; ^5^ GlyProVac ApS, Odense, Denmark; ^6^ Norwegian National Advisory Unit on Tropical Infectious Diseases, Department of Medicine, Haukeland University Hospital, Bergen, Norway

**Keywords:** enterotoxigenic *Escherichia coli*, immunogenicity, vaccine development, protein glycosylation, IgA, SslE, YghJ

## Abstract

Efforts to develop broadly protective vaccines against pathogenic *Escherichia coli* are ongoing. A potential antigen candidate for vaccine development is the metalloprotease YghJ, or SslE. YghJ is a conserved mucinase that is immunogenic, heavily glycosylated, and produced by most pathogenic *E. coli*. To develop efficacious YghJ-based vaccines, there is a need to investigate to what extent potentially protective antibody responses target glycosylated epitopes in YghJ and to describe variations in the quality of YghJ glycosylation in the *E. coli* population. In this study we estimated the proportion of anti-YghJ IgA antibodies that targeted glycosylated epitopes in serum and intestinal lavage samples from 21 volunteers experimentally infected with wild-type enterotoxigenic *E. coli* (ETEC) strain TW10722. Glycosylated and non-glycosylated YghJ was expressed, purified, and then gycosylation pattern was verified by BEMAP analysis. Then we used a multiplex bead flow cytometric assay to analyse samples from before and 10 days after TW10722 was ingested. We found that 20 (95%) of the 21 volunteers had IgA antibody responses to homologous, glycosylated YghJ, with a median fold increase in IgA levels of 7.9 (interquartile range [IQR]: 7.1, 11.1) in serum and 3.7 (IQR: 2.1, 10.7) in lavage. The median proportion of anti-YghJ IgA response that specifically targeted glycosylated epitopes was 0.45 (IQR: 0.30, 0.59) in serum and 0.07 (IQR: 0.01, 0.22) in lavage. Our findings suggest that a substantial, but variable, proportion of the IgA antibody response to YghJ in serum during ETEC infection is targeted against glycosylated epitopes, but that gut IgA responses largely target non-glycosylated epitopes. Further research into IgA targeting glycosylated YghJ epitopes is of interest to the vaccine development efforts.

## Introduction

*Escherichia coli* are versatile bacteria, with some variants colonizing human hosts as commensals, while others possess virulence factors that can cause everything from mild to lethal intestinal or extraintestinal diseases (1). The effort to develop vaccines against pathogenic *E. coli* has been ongoing for several decades (2, 3), and several different protein virulence factors are currently being evaluated for use in vaccines against these pathogens (4, 5). A relatively recently identified promising vaccine target is the virulence factor YghJ, also known as SsIE, which is a large metalloprotease secreted through the Type II secretion system (T2SS) of most pathogenic *E. coli* (6–8). Through the action of its M60-like aminopeptidase domain (9), YghJ can erode the protective mucus layer that protect mucosal membranes in humans by degrading MUC2, MUC3, and MUC5AC proteins, thus allowing *E. coli* to reach the epithelial cell surface and start colonization (8, 10). There is also evidence that YghJ help mediate *E. coli* biofilm formation both during colonization (11) as well as when surviving outside the host (12). In enterotoxigenic *E. coli* (ETEC), YghJ is secreted through T2SS, which also mediates secretion of the ETEC heat-labile toxin (LT) (13). Antibodies against YghJ have been shown to impair LT delivery to target cells *in vitro*, indicating that YghJ may facilitates diarrhea induction during ETEC infection (10).

In mouse experiments, immunization with recombinantly produced YghJ protected animals against extraintestinal pathogenic *E. coli* bacteremia, intranasal immunization with YghJ impaired colonization of pathogenic *E. coli*, and subcutaneous YghJ immunization protected against sepsis (14). Natural and experimental ETEC infections in humans appear to consistently generate strong cell- and systemic antibody-mediated immune responses against YghJ (6, 15–17). When used in vaccines, it is expected that the most effective anti-YghJ immune responses would be mediated through secretion of mucosal IgA antibodies capable of neutralizing the mucinase activity or to disrupt its role in forming biofilms, thus limiting the bacteria’s access to and ability to colonize the epithelial cells.

The YghJ secreted by pathogenic *E. coli* has been shown to be heavily glycosylated by O-linked glycosylation (18). This glycosylation entails the attachment of one or more carbohydrate molecules (glycans) to many of the protein’s serine and threonine amino acid residues (19). O-linked glycosylation of surface-exposed proteins has been found to be widespread in the *E. coli* population and is more comprehensive in pathogenic than in commensal *E. coli* (18). Such post-translational protein modifications usually change proteins’ phenotypic properties and can affect the pathogen’s ability to adhere to, colonize, or penetrate the host tissue (20–22).

Since glycosylation may affect the antigenic properties of proteins, glycosylation should also be taken into consideration when designing subunit vaccines based on proteins that harbor such glycosylations. The importance of this has been well documented in the work with vaccine candidates against HIV-1, where glycosylated epitopes seem to elicit more broadly neutralizing antibodies than linear peptide epitopes (23, 24). Similarly, for developing vaccines against Tuberculosis, Romain et al. (25) found that de-glycosylating the protein-based vaccine antigens resulted in substantially poorer T lymphocyte responses, suggesting that immune responses to subunit vaccines may be improved if vaccine antigens have the same glycosylation as the native proteins produced by the target pathogen.

A protective effect from immunization with YghJ is likely to be mediated by protective antibodies at the gut or urethal mucosal surfaces inhibiting mucinase function or biofilm formation, limiting the entry of toxins or invading bacteria. Given that native YghJ is heavily glycosylated, it is expected that immunizing with native, glycosylated YghJ antigens would give different immune responses than immunization with antigens that lack this glycosylation. However, the proportion of antibodies targeting glycosylated YghJ epitopes after natural pathogenic *E. coli* infection is not known.

To explore the effect that glycosylation has on the quality of immune responses to YghJ, in this study we evaluate human systemic and gut anti-YghJ IgA antibody responses and the extent to which they actually target glycosylated epitopes of YghJ produced during infections with pathogenic *E. coli*. To achieve this, we produced native secreted glycosylated YghJ and recombinant non-glycosylated YghJ and used a multiplex bead-based flow cytometric immunoassay to estimate anti-YghJ IgA responses against these YghJ variants. In addition, we have also selectively neutralized antibodies targeting non-glycosylated YghJ epitopes to estimate the proportion of anti-YghJ IgA that specifically targeted glycosylated epitopes.

## Materials and Methods

### Experimental ETEC Infection Study

Details of the experimental ETEC infection study have been described earlier by Sakkestad et al. (16). Briefly, 21 healthy adult volunteers aged between 18 and 40 years, who were presumed to be immunologically naïve to ETEC, were experimentally infected with ETEC strain TW10722 by ingesting doses ranging from 1 × 10^6^ to 1 × 10^10^ colony forming units (CFU). The infection was cleared 5 days after dose ingestion, or within 24 hours of experiencing severe symptoms, by treatment with ciprofloxacin. The volunteers were considered to have diarrhea if they passed 1 loose or liquid stool weighing ≥ 300 g, or ≥ 2 loose or liquid stools combinedly weighing ≥ 200 g during any 48-hour period within 120 hours after dose ingestion. Ten of the 21 volunteers developed diarrhea (16).

ETEC strain TW10722 (O115:H5; GenBank BioProject: PRJNA59745) was isolated in 1997 in Guinea-Bissau from a 15-month-old child who was suffering from acute diarrhea. The strain encodes the two ETEC colonization factors coli surface antigen 5 (CS5) and CS6. It also encodes the human variant of the heat-stable enterotoxin (STh), but not the heat-labile toxin (LT).

### Specimen Collection and Preparation

In the present study, we use serum samples from these 21 volunteers collected on the day of dose ingestion and 10 days after, as well as intestinal lavage samples collected a few weeks before dose ingestion and 10 days after.

To obtain intestinal lavage specimens, the volunteers drank a polyethylene glycol-based laxative (Laxabon; Karo Pharma AB, Stockholm, Sweden) until the stools were clear and watery. After mixing with EDTA-free cOmplete Protease Inhibitor Cocktail (Roche, Basel, Switzerland), samples were immediately stored at -70°C until the experimental infection study had ended. For the current analyses, aliquots of these lavage specimens were thawed on ice, centrifuged at 16,000 × g for 3 min, and the supernatant was subsequently filtered successively through 1 µm, 0.45 µm, and 0.22 µm pore size syringe filters before being stored at -70°C until use.

### Glycosylated YghJ Production

To obtain YghJ from TW10722 that could be purified but that was still fully glycosylated, we inserted a DNA sequence encoding the 3xFLAG peptide tag immediately after the native YghJ gene on the TW10722 chromosome and utilized this tag to affinity-purify YghJ from the bacterial culture supernatant. In the procedure, which was based on the recombinational tagging protocol described by Uzzau et al. (26), a PCR product was generated by using the 3xFLAG sequence and kanamycin resistance gene in pSUB11 as a template and the primer pair GPV128+GPV129 (**Table 1**). After purification, the PCR product was electroporated into TW10722 by using a Bio-Rad gene pulser (pulse parameters: 1.80 kV, 25 µF, and 200 Ω). Transformants were selected on Luria Bertani (LB) agar plates containing 40 µg/ml kanamycin. Sanger sequencing based on primer pairs GPV127+GPV17 and GPV67+GPV147 (**Table 1**) were used to verify that the 3xFLAG sequence was correctly inserted.

**Table 1:** Sequences of DNA primers used for cloning and testing YghJ constructs.

Primer ID	Sequence	Primer name
GPV17	AGCAGCGGAA TATTGTCACG TAT	yghJ rv
GPV67	GAAGGAATGG GCAGAGAAAA ACT	yghJ fw segment 7
GPV127	TCGTTAATAT CATCCGGCTT CAT	yghJ fw
GPV128	AAGCTGCCGA AACCGGAACA GGGACCGGAA ACCATTAACA AGGTTACCGA GCATAAGATG TCTGTCGAGG ACTACAAAGA CCATGACGG	yghJ 3xFLAG fw
GPV129	TAAGCTGGCG CAACCCGGTG CGCCTTATTT CATGCCGGAT GCGGCGTGAA CGCCTTATCC GGCATACAGG ACATATGAAT ATCCTCCTTA G	yghJ 3xFLAG rv
GPV130	ACTTAGATTC AATTGTGAGC CACCATAAGG AGTTTTATAA ATGAATAAGA AATTTAAATA TAAGA	ETEC TW10722 IPTG SD yghJ fw1
GPV131	TAGCTACTCG AGGGCAAAAA GAGTGTTGAC TTGTGAGCGG ATAACAATGA TACTTAGATT CAATTGTGAG CCACCAT	ETEC TW10722 IPTG SD yghJ fw2
GPV132	TATCATGATC TTTATAATCA CCGTCATGGT CTTTGTAGTC CTCGACAGAC ATCTTATGCT CGGTAAC	ETEC TW10722 yghJ FLAG rv1
GPV133	TAGCTATCTA GATTACTATT TATCGTCGTC ATCTTTGTAG TCGATATCAT GATCTTTATA ATCACCGTCA T	ETEC TW10722 yghJ FLAG rv2
GPV 97	TAGCTAGCTC TAGTTACTAT TTATCGTCGT CATCTTTG	FLAG rv
GPV147	CAGTCATAGC CGAATAGCCT	K1 oligo, Wanner

To purify glycosylated YghJ from this modified TW10722 strain, the strain was grown in M9 minimal medium (27) supplemented with 0.2% glucose, 0.4% casamino acid, and 40 μg/ml kanamycin. The culture was grown at 37°C to an OD_600_ of 2.5 before harvesting the YghJ-containing supernatant by centrifugation at 15,250 × g at 2°C for 20 min. The supernatant was sterile filtered (0.22 μm pore size) before NaCl and Triton X-100 were added to 200 mM and 0.01% final concentrations, respectively. ANTI-FLAG M2 Affinity Gel (Product no.: A2220; Sigma-Aldrich, St. Louis, MO) agarose beads were added to the supernatant and incubated shaken overnight at 4°C. After sterile filtration, the recovered beads were washed twice with FLAG Sup wash buffer I (phosphate -buffered saline [PBS], pH 7.6, containing 400 mM NaCl, 0.1% Triton X-100, and 1 mM EDTA) and once with FLAG Sup wash buffer II (PBS, pH 7.6, containing 400 mM NaCl, 0.01% Triton X-100, and 1 mM EDTA). The glycosylated YghJ was eluted by incubating in Elution buffer (500 mM arginine, 500 mM NaCl, pH 3.5), after which 1 M Tris base was added until the pH reached 7.6. Eluates were spin filter concentrated before overnight dialysis at 4°C against PBS, pH 7.6, containing 0.1% Triton X-100, which helps to keep the glycosylated YghJ in solution.

### Non-Glycosylated YghJ Production

To produce non-glycosylated YghJ, we cloned *yghJ* and the appended 3xFLAG sequence generated above into an expression vector and produced it recombinantly in a modified *E. coli* MG1655 strain called MG1655Δ*hldE*, which contains a non-functioning *hldE*. HldE is responsible for synthesizing the heptose glycans that *E. coli* use for protein glycosylation (28). We first amplified the *yghJ* sequence and 3xFLAG sequence from the modified TW10722 described above by using primer pairs GPV130+GPV132 (**Table 1**). This PCR product was then further amplified by using primer pairs GPV131+GPV133 to generate a product that was subsequently digested with XhoI and XbaI and ligated into the expression vector pXG-0 (29), creating pGPV106. Before transforming pGPV106 into *E. coli* MG1655ΔhldE, we sequenced it to ensure that *yghJ* and the 3xFLAG sequence were correctly inserted (**Supplementary Table S1**).

To produce the non-glycosylated YghJ, this strain was grown at 37°C in 10 L LB medium supplemented with 40 μg/ml chloramphenicol and 1 mM isopropyl-β-D-1-thiogalactopyranoside (IPTG) until reaching an OD_600_ of 2.5, before we harvested the YghJ-containing cells by centrifugation at 15250 × g for 15 min at 2°C. Cell pellets were resuspended in PBS, pH 7.6, containing 500 μg DNase I before being passed three times through a French Press at 2.2 kbar. The resulting lysate was cleared by ultracentrifugation at 125,000 × g for three hours at 4°C in a swinging-bucket rotor before diluting the resulting supernatant to 1 L with PBS, pH 7.6, containing NaCl, Triton X-100, and EDTA to final concentrations of 600 mM, 0.01%, and 1 mM, respectively. The non-glycosylated YghJ was then purified, concentrated, and dialyzed as described above for the glycosylated YghJ production, with the exception that the FLAG Sup wash buffer I and II contained 600 mM instead of 400 mM NaCl.

### Protein Testing

The purified proteins were quantified by using the BCA Micro assay (Thermo Fisher Scientific, Waltham, MA). We confirmed that the purified proteins contained YghJ by performing native and denaturing western blotting. In these assays, 50 ng native or denatured YghJ proteins were separated on NuPAGE 4 to 12% gradient Bis-Tris polyacrylamide gels (Thermo Fisher Scientific) by using native PAGE or SDS-PAGE, respectively, together with the SeeBlue Plus2 Pre-stained Standard (Thermo Fisher Scientific) molecular weight marker, before being transferred to a polyvinylidene difluoride (PVDF) membrane. To obtain a clear separation of YghJ in the native PAGE, we mixed the protein in SDS native loading buffer (60 mM Tris-Cl, pH 6.8, 10% glycerol, 0.005% bromophenol blue) and used an MES-based running buffer that contained small amounts of SDS (50 mM MES, 50 mM Tris-base, 0.01% SDS, pH 7.3). We found that the addition of 0.01% SDS greatly improved the band resolution in these assays.

To detect FLAG-tagged YghJ, the membranes were incubated in PBS containing 1% skimmed milk powder and 0.05% Tween-20 as follows: 1 hour without any additives, 1 hour with monoclonal anti-FLAG M2 mouse antibodies (Product no.: F3165; Sigma-Aldrich), and 1 hour with HRP-conjugated rabbit Anti-mouse IgG (Product no.: P0260; Dako Denmark AS, Næstved, Denmark). The blot images were captured on a GE Amersham Imager 680 (GE Healthcare, Chicago, Il) after wetting with Immobilon Forte Western HRP substrate (Merck KGaA, Darmstadt, Germany). When testing for YghJ-specific antibodies in serum from the infected volunteers, we instead first incubated the membranes in PBS containing 3% skimmed milk powder and 0.05% Tween-20, before adding diluted volunteer serum, and we used HRP-conjugated polyclonal rabbit anti-Human IgA, IgG, IgM, Kappa, Lambda antibody (Product no.: P0212; Dako Denmark AS) as the secondary antibody.

To evaluate whether the native YghJ was actually glycosylated, we performed beta-elimination of O-linked glycans followed by Michael-addition of a phosphonic acid derivative (BEMAP) analysis to check the extent of glycosylation on the protein’s serine and threonine residues, as previously described (18). Briefly described, the BEMAP chemistry catalyses the substitution of glycans attached to peptides with a phosphorylated tags. Following the conversion, the BEMAP protocol includes an enrichment step based on TiO2 beads which selectively allows one to isolate the phosphopeptides. These peptides are analyzed by mass spectrometry. The downstream filtering of data is carried out using the Thermo Proteome Discover software tool 2.4 and only phosphopeptides are selected for final evaluation. It should be noted that the BEMAP method can only determine if a site is likely to be modified or not. Therefore, the method will not be able to evaluate what proportion of the proteins were glycosylated at any given site.

### Protein Bead Coupling

The proteins, including glycosylated YghJ (gYghJ) and non-glycosylated YghJ (nYghJ) were covalently coupled to 4 µm Cyto-Plex carboxylated beads (Thermo Fisher Scientific, Waltham, MA) of different fluorescence levels. Since the YghJ protein solutions contained Triton X-100, which may interfere with and reduce the efficiency of coupling the proteins directly onto these polystyrene beads, we first coupled long polyethylene glycol linkers to the beads and then coupled the proteins to these linkers. Both coupling reactions were done by using carboxyl-to-amine crosslinking chemistry based on N-(-dimethylaminopropyl)-N’-ethyl carbodiimide HCl (EDC) and N-hydrosulfosuccinimide (Sulfo-NHS).

The wells of a MultiScreen HTS filter plate (Merck KGaA) were wetted with 100 µL MES buffer (50 mM 2-[N-morpholino] ethanesulfonic (MES) acid, pH 5.5) followed by centrifugation at 300 × g for 45 sec at room temperature. All remaining centrifugations were done at a lower 50 × g for 45 sec at room temperature to minimize the risk of beads sticking to the filter, and during the first wash in each step described below, we also scraped the tip of the pipet along the edges of the well-bottom when mixing to ensure complete resuspension of the beads. Approximately 8 million beads diluted in 50 µl MES buffer were added to the wells, followed by centrifugation, wash in 200 µl MES buffer, and centrifugation. A mix of 160 µL MES buffer, 20 µL MES buffer containing 50 mg/mL freshly prepared EDC, and 20 µL MES buffer containing 50 mg/mL freshly prepared Sulfo-NHS were added and mixed with the beads. After incubating at room temperature for 20 minutes on a microplate shaker (all shaking was done at 600 rpm, 3 mm Ø), the beads were washed twice with 200 µL MES buffer. We then added 200 µl MES buffer containing 2 mg of the PEG linker (Poly [ethylene glycol] 2-aminoethyl ether acetic acid of 2.1 kDa average molecular weight [Product no. 757888-100MG; Sigma-Aldrich]) and incubated for 2 h at room temperature on a shaker, followed by three washes. To couple the proteins to the carboxylated ends of the PEG linkers, we repeated the last steps above, including adding fresh EDC and Sulfo-NHS, incubating and washing, before adding 18 µg of non-glycosylated or glycosylated YghJ. The protein concentrations were re-measured by using the BCA Micro assay prior to the addition. After overnight incubation on a shaker at 4°C, the beads were washed twice with PBS, pH 7.4, and resuspended in 400 µL Assay buffer (PBS, pH 7.4, 1% bovine serum albumin [BSA], and 0.05% Tween-20), before the bead concentration was determined by using a Bürker counting chamber. The beads were then stored at 4°C and were used within two months period.

### Bead-Based Anti-YghJ Antibody Assay

In the bead-based antibody assays, we pooled beads coupled with non-glycosylated YghJ (nYghJ) and glycosylated YghJ (gYghJ). For estimating fold-changes, serum samples were diluted 1:50 in Assay buffer, lavage samples were diluted 1:2 in 2X Assay buffer, and the secondary antibody (Alexa fluor 488 Affinipure Goat Anti-human serum IgA antibody [Jackson ImmunoResearch, West Grove, PA]) was diluted 1:200 (for lavage) or 1:400 (for serum) in Assay buffer and kept on ice before use.

After wetting the wells of a MultiScreen HTS HV filter plate by adding 100 µl Assay buffer followed by centrifugation at 300 × g at room temperature for 45 sec, we added 5000 beads of each gYghJ and nYghJ to 50 µl Assay buffer and combined this with 50 µl diluted serum or lavage samples in the filter plate. After 30 min incubation at room temperature on the microplate shaker, the beads were washed twice, as described for the wash steps for the bead-preparations in the previous section, with 200 µl Assay buffer. We subsequently incubated the beads shaken at room temperature for 30 min in 50 µL secondary antibody, followed by two washes with 200 µL Assay buffer, and resuspension of the beads in 200 µl Assay buffer, followed by analysis on an LSR Fortessa flow cytometer (BD Life Sciences, Franklin Lakes, NJ).

Single beads were identified and gated by using forward and side light scatter readings. The single beads were then gated on fluorescence emission intensity at 700 nm to identify the bead population and, therefore, YghJ variant, and at 520 nm to measure the amount of IgA bound to each of the beads. The median fluorescence intensity (MFI) for each protein, or bead population, was estimated by using FlowJo, version 10.4.2 (BD Life Sciences).

The estimated MFI for each protein in each assay was normalized by interpolating from standard curves that had been created by duplicate dilution series of a high-titer sample and setting the highest MFI reading to 10000 arbitrary units (AUs) (**Supplementary Table S2**).

### Total IgA Quantitation

The lavage samples may have different levels of IgA as a result of variations in antibody secretion during the intestinal lavage sample collection, and the amount of lavage fluid the volunteers consumed. To be able to compensate for different IgA levels in these samples, we measured the IgA concentration in all lavage samples by using the Human IgA Flex Set Kit (BD Life Sciences), as described by the producer. We then normalized the anti-YghJ assay results, measured in AUs, by dividing by the estimated total IgA concentration in the given sample to obtain normalized arbitrary units (nAUs) (**Supplementary Table S3**).

### IgG and IgM Depletion in Serum Samples

To accurately measure the glycosylation-specific anti-YghJ IgA response in serum samples, we first removed IgG and IgM antibodies since these appeared to compete with IgA binding to YghJ, and thereby disturbed comparability between samples preincubated or not preincubated with YghJ variants in the glycosylation-specificity assay (**Supplementary Table S4**).

The removal of IgG and IgM from serum was done by pre-incubating diluted serum with anti-human IgG and anti-human IgM antibody-coupled agarose beads (Sigma-Aldrich; Product nos. A9935 and A3316, respectively) and using the resulting serum in our Glycosylation specificity assay, as described in the next section. Specifically, we used wide-bore pipet tips to transfer agarose beads to wash the beads once in 1 mL Assay buffer followed by centrifugation at 1000 × g for 1 minute and discarding an equal amount of supernatant. We then added serum that had been diluted 1:50 in Assay buffer and incubated the plate on a shaker overnight at 4°C. Following centrifugation of the filter plates at 300 × g for 45 seconds, the resulting filtered samples were further diluted 1:2 in Assay buffer (1:100 final dilution) immediately before performing the glycosylation specificity assay.

Prior to the treatment, we had optimized the bead volumes needed to effectively remove IgG and IgM from the serum samples. Based on those tests, we added 17 µL anti-IgG and 6 µL anti-IgM beads per microliter 1:50 diluted serum.

### Glycosylation Specificity Assay

To evaluate the degree to which anti-YghJ IgA responses specifically target glycosylated YghJ epitopes, we designed and performed a glycosylation specificity assay. In this assay, we pre-incubated the serum or lavage samples with non-glycosylated YghJ so that anti-YghJ antibodies that do not target glycosylated epitopes have been bound to free YghJ and are therefore less likely to bind to bead-bound gYghJ in the subsequent bead-based anti-YghJ antibody assay. By comparing the estimated anti-YghJ IgA levels in these samples with those of the untreated samples and of the samples that had been treated with glycosylated YghJ, we can calculate the proportion of the anti-YghJ IgA response that target epitopes specific for glycosylated YghJ. This is done by first subtracting the assay background (i.e., estimated antibody levels after pre-incubating the sample with glycosylated YghJ) from the antibody levels estimated from untreated and non-glycosylated YghJ-treated samples, followed by dividing the antibody levels estimated from non-glycosylated YghJ-treated samples with those of the untreated samples. When optimizing the conditions for obtaining the assay background levels, we found that when pre-incubating with a combination of glycosylated and non-glycosylated YghJ we did not obtain any lower background level estimates than when pre-incubating with glycosylated YghJ alone (**Supplementary Table S5**). This suggests that the glycosylated YghJ also expose most, or all of the epitopes presented by the non-glycosylated YghJ and that pre-incubating with glycosylated YghJ is sufficient to remove most or all anti-YghJ IgA antibodies.

In this assay, we prepared 50 µL aliquots of serum that had been IgG- and IgM-depleted and diluted 1:100 in assay buffer or native intestinal lavage diluted 1:2 in 2X assay buffer in 3 Eppendorf tubes and added 1 µg nYghJ, 1 µg gYghJ, and 1 µL assay buffer, respectively, to the three tubes before incubating them shaken at room temperature for 30 min. The 1 µg YghJ added to these tubes represents an excess of > 300 times over the number of YghJ proteins coupled to the beads used in our bead-based assay (**Supplementary Table S5**) and adding 1 µg YghJ was found to be a good compromise to effectively remove anti-YghJ antibodies while using a minimum of purified YghJ. After incubation, the treated samples were used directly in the bead-based anti-YghJ antibody assay as described above.

### Statistical Analysis

For all statistical analyses and for preparing graphs, we used Prism, version 9 (GraphPad Software, San Diego, CA), and we considered p-values ≤ 0.05 to be statistically significant. To test for differences in IgA levels between day-0 and day-10 serum and lavage samples, we used Wilcoxon matched-pairs signed ranks test. To test for differences in IgA levels between volunteers who developed diarrhea and those who did not, we used the Mann-Whitney U test. To assess the correlation in IgA levels between and within lavage and serum samples, we calculated Pearson correlation coefficients.

### Ethical Approval

All volunteers signed written informed consent to participate in the study and could leave at any point at their discretion. The experimental infection study (NCT02870751 at ClinicalTrials.gov) was approved by the Regional Committee for Medical and Health Research Ethics, Health Region West (REC-West; case number 2014/826).

## Results

### YghJ Preparations and Testing

**Figure 1:**
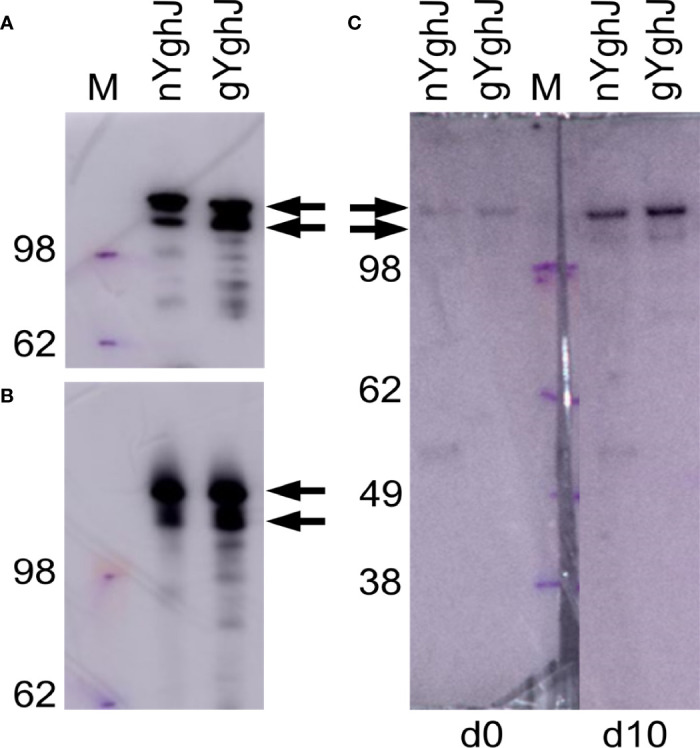
Western blots of non-glycosylated (nYghJ) and glycosylated (gYghJ) YghJ. **(A, B)** shows blots where YghJ was detected by targeting the 3xFLAG peptide that trail YghJ. In **(C)**, the blots were incubated with serum from volunteer EV01 before (d0) and 10 days after (d10) dose ingestion, followed by detection of any bound IgA, IgG, or IgM antibodies. The marker (M) band sizes are listed on the left-hand side. Arrows indicate expected sizes of YghJ. The blots were based on denatured **(A, C)** and native gels **(B)**.

We found that the non-glycosylated YghJ and the native, glycosylated YghJ had the expected sizes when separated by native and denaturing gel electrophoresis and detected by anti-FLAG antibodies (**Figure 1**). This indicated no gross conformational differences between our non-glycosylated and glycosylated YghJ preparations.

To test that our volunteers developed anti-YghJ antibody responses that could recognize our purified YghJ, we analyzed serum from our first volunteer (EV01) in a denaturing western blot against our YghJ extracts. We found that the serum collected both before and 10 days after dose ingestion contained antibodies that targeted the correctly sized protein band, and that the day 10 serum antibody levels appeared to be substantially higher than the pre-ingestion serum (**Figure 1C**), suggesting that our purified YghJ are recognized by anti-YghJ antibodies produced by the volunteers. There appeared to be no other strong bands in those blots, suggesting that the results of our antibody assays will reflect antibody levels against YghJ and not against any other proteins potentially contaminating our protein preparations.

The BEMAP analyses showed that YghJ in our glycosylated YghJ preparation indeed was glycosylated, with 50 of the serine and threonine residues being O-glycosylated (**Supplementary Table S6**).

### Serum and Lavage Samples

In these analyses, we included a set of 21 serum and lavage samples taken before (day 0), and 10 days (day 10) after the dose was ingested. Ten (48%) of the 21 volunteers developed diarrhea. In our analyses, we included 19 pairs of lavage samples. Lavage samples from two volunteers were excluded because we could not detect any IgA in one sample of each pair. Median total IgA concentration in the lavage samples was 0.7 (IQR: 0.6, 1.9) mg/mL in the day 0 samples and 1.4 (IQR: 0.7, 2.2) mg/mL in the day 10 samples, with the difference not being statistically significant (**Supplementary Table S3**).

### Changes in Anti-YghJ IgA Levels Following ETEC Infection

To evaluate the strength of the IgA antibody response to YghJ following the experimental infection, we used our Bead-based anti-YghJ antibody assay to estimate the change in levels of YghJ-specific IgA antibodies in serum and lavage from day 0 to day 10.

**Figure 2:**
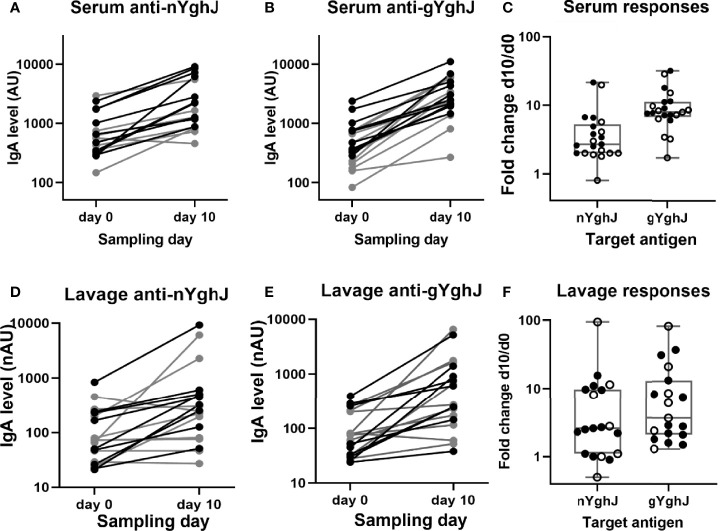
Anti-YghJ IgA antibody level changes in serum and lavage from before and 10 days after dose ingestion. Graphs show changes in serum IgA targeting non-glycosylated YghJ **(A)** and glycosylated YghJ **(B)** from before (day 0) and 10 days after ingesting ETEC, as well as the corresponding fold changes in serum **(C)**. The figures also show changes in lavage IgA targeting non-glycosylated YghJ **(D)** and glycosylated YghJ **(E)** from before (day 0) and 10 days after ingesting ETEC, as well as the corresponding fold changes in lavage **(F)**. Grey lines (in **A, B, D, E**) and open circles (in **C, F**) represent volunteers who did not develop diarrhea. Correspondingly, black lines and filled circles represent volunteers who developed diarrhea. Line in boxes represents median values and boxes the values between 25th and 75th percentiles. IgA levels are expressed as arbitrary units (AU) for serum and normalized arbitrary units (nAU) for lavage.

We found that the anti-YghJ IgA levels in serum rose significantly from day 0 to day 10, both when tested against nYghJ (median 564 [IQR: 360, 770] AU on day 0 to 1653 [IQR: 1181, 5553] AU on day 10; *p* < 0.001; **Figure 2A**) and against gYghJ (median 299 [IQR: 208, 666] AU on day 0 to 2495 [IQR: 2042, 5042] AU on day 10; *p* < 0.001; **Figure 2B**). The corresponding median fold change was 2.7 (IQR: 2.0, 4.9) for nYghJ and 7.9 (IQR: 7.1, 11.1) for gYghJ (**Figure 2C**), giving a statistically significant difference (p < 0.001) in fold change between nYghJ and gYghJ. Defining responders to be volunteers who had a ≥ 2.0-fold increase in anti-YghJ IgA levels, we found that 18 of the 21 volunteers (86%) were responders when tested against nYghJ, while 20 of 21 (95%) were responders when tested against gYghJ.

Correspondingly, for the lavage samples, we found significant increases in median anti-nYghJ IgA levels from day 0 to day 10, both when tested against nYghJ (median 72 [IQR: 37, 213] nAU on day 0 to 250 [IQR: 103, 497] nAU on day 10; *p* < 0.001; **Figure 2D**) and against gYghJ (median 75 [IQR: 32, 207] nAU on day 0 to 271 [IQR: 158, 1151] nAU on day 10; *p* < 0.001; **Figure 2E**). The corresponding median fold change was 2.6 (IQR: 1.1, 9.7) for nYghJ and 3.7 (IQR: 2.0, 10.7) for gYghJ (**Figure 2F**), with no statistically significant difference in fold change between nYghJ and gYghJ. Thirteen of the 19 volunteers (68%) were responders when testing against nYghJ, while 14 of 19 (74%) were responders when testing against gYghJ.

The fold changes in anti-nYghJ IgA concentrations correlated well with the corresponding anti-gYghJ IgA fold changes both in serum (r= 0.92, p < 0.001) and in lavage (r= 0.90, p < 0.001) (**Supplementary Figure 1**). There was no significant correlation between anti-nYghJ IgA fold-changes in serum and lavage samples (r = 0.10; p = 0.68), and neither between anti-gYghJ IgA (r = 0.09; p = 0.72) (**Supplementary Figure 2**). Neither in serum nor in lavage, did we find any statistically significant difference in anti-nYghJ IgA or anti-gYghJ IgA fold changes between volunteers who developed diarrhea compared to those who did not.

### Proportion of Anti-YghJ IgA Targeting Glycosylated Epitopes

Here we estimated the proportion of anti-YghJ IgA antibodies that target glycosylated epitopes on YghJ. As described in Methods, the proportions were estimated by comparing the anti-YghJ levels after pre-incubating with buffer (giving total anti-YghJ IgA levels) with the anti-YghJ levels after pre-incubating with non-glycosylated YghJ (giving anti-YghJ IgA levels targeting glycosylated epitopes) after subtracting the anti-YghJ levels after pre-incubating with glycosylated YghJ (giving the assay background levels). To obtain accurate estimates, we focused these analyses on the day 10 samples and only on samples that had anti-YghJ levels ≥ 100 AU (for serum) and ≥ 100 nAU (for lavage), thus ending up with 16 serum and 11 lavage samples. Prior to analyses, we removed IgG and IgM from the serum samples by pre-incubating the serum with immobilized anti-IgG and -IgM antibodies to ensure that the IgG and IgM did not compete with IgA for binding to YghJ.

**Figure 3:**
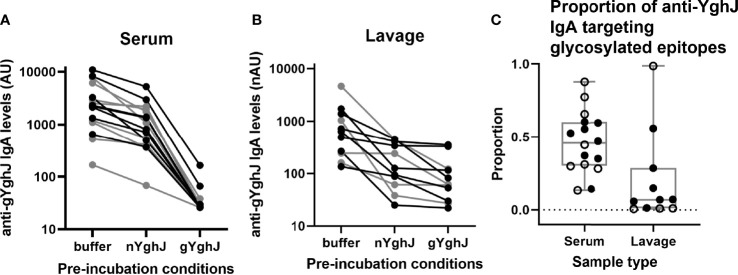
Glycosylated epitope specificity in serum and lavage. Figures show anti-gYghJ antibody levels in serum after pre-incubation with buffer or nYghJ, or gYghJ **(A)** and anti-gYghJ antibody levels in lavage after pre-incubation with buffer or nYghJ, or gYghJ **(B)**. Graph **(C)** shows the proportion of anti-gYghJ-specific antibodies out of total anti-nYghJ and anti-gYghJ specific IgA antibodies in serum and lavage. Grey lines and open circles represent volunteers who did not develop diarrhea. Correspondingly, black lines and filled circles represent volunteers who developed diarrhea. Line in the boxes represents median values and boxes the values between 25th and 75th percentiles. The upper and lower whiskers limit 95% of measured values. IgA levels are expressed as AU for serum and normalized arbitrary units (nAU) for lavage.

In serum, the median anti-gYghJ IgA antibody level was 2266 (IQR: 1170, 3992) AU (**Figure 3A**, column “buffer”), the median IgA level for antibodies targeting glycosylated YghJ epitopes was 943 (IQR: 477, 1813) AU (**Figure 3A**, column “nYghJ”), and the median assay background was 27 (IQR: 26, 30) AU (**Figure 3A**, column “gYghJ”). The median proportion of anti-YghJ IgA antibodies targeting glycosylated epitopes in these 16 volunteers was 0.45 (Range: 0.13, 0.88; IQR: 0.30, 0.59) (**Figure 3C**, column “Serum”).

In lavage, the median anti-gYghJ IgA antibody level was 638 (IQR: 254, 1,217) nAU (**Figure 3B**, column “buffer”), the median IgA level for antibodies targeting glycosylated YghJ epitopes was 126 (IQR: 74, 373) nAU (**Figure 3B**, column “nYghJ”), while the median assay background was 62 (IQR: 41, 119) nAU (**Figure 3B**, column “gYghJ”). The median proportion of anti-YghJ IgA antibodies that target glycosylated epitopes in these 11 volunteers was 0.07 (Range: 0.01, 0.98; IQR: 0.01, 0.22) (**Figure 3C**, column “Lavage”). While all volunteers had some levels of glycosylation-specific anti-YghJ IgA in their serum, 7 of the 11 (64%) appeared to have little or no glycosylation-specific anti-YghJ IgA in their lavage samples. There did not seem to be a clear correlation between having diarrhoea and the proportion of anti-YghJ IgA targeting glycosylated epitopes in serum or lavage (**Figure 3C**).

**Figure 4:**
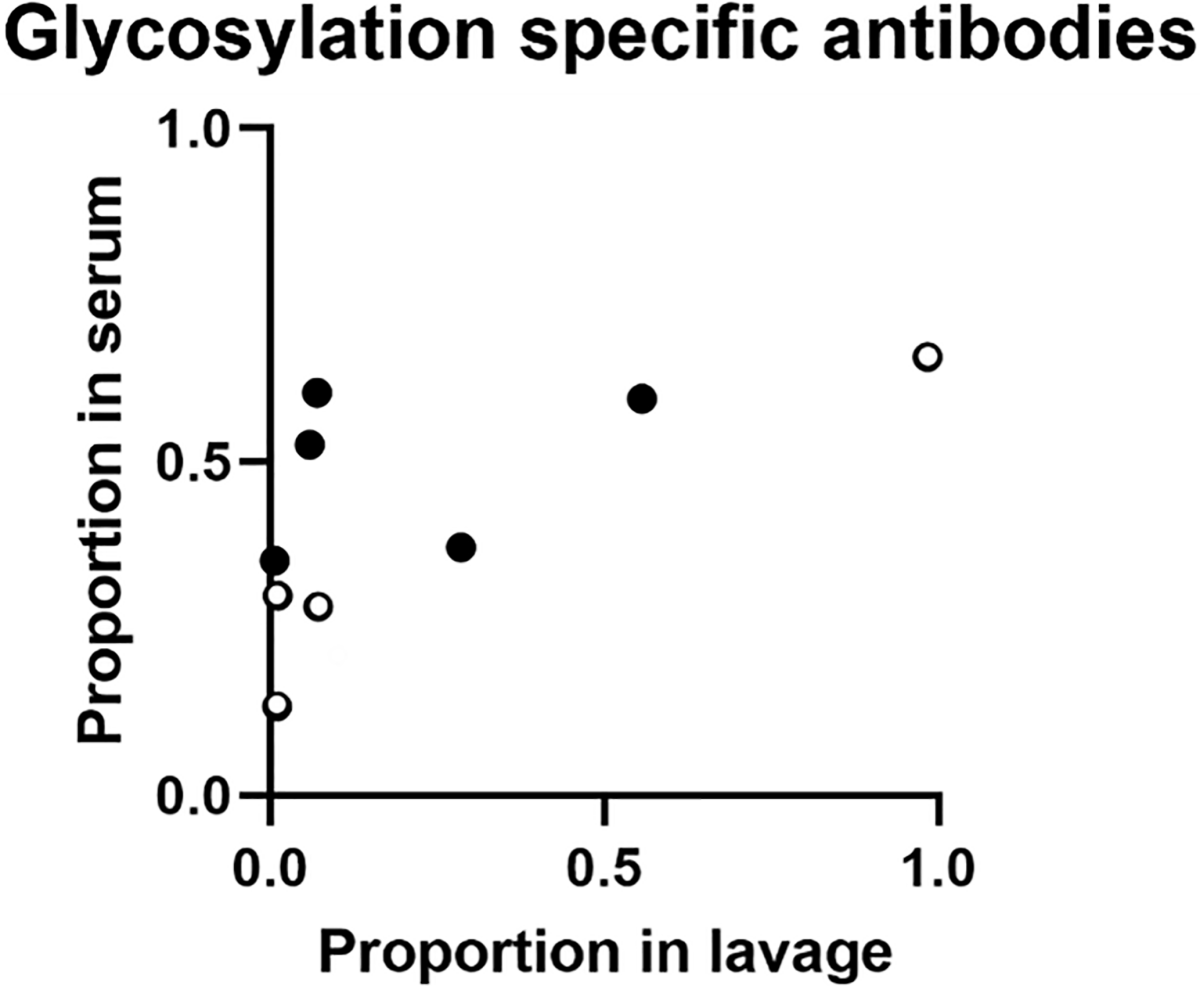
Proportion of glycosylation specific anti-YghJ IgA in lavage and serum samples. Open circles represent volunteers who did not develop diarrhea and filled circles represent volunteers who developed diarrhea.

Comparing results of samples from the 9 volunteers who contributed with both serum and lavage samples in these analyses, the proportion of IgA targeting glycosylated YghJ epitopes in serum did not seem to be strongly correlated to the corresponding proportion found in the volunteers’ lavage (*r* = 0.65, *p* = 0.057; **Figure 3**). This borderline correlation appeared mainly to be an effect of some volunteers with considerable proportions of glycosylation specific anti-YghJ IgA in serum, but little or none in their lavage sample (**Figure 4**).

## Discussion

We found that infection with ETEC TW10722 elicited substantial IgA antibody responses against YghJ in most volunteers. We further found that relatively large proportions of anti-YghJ IgA antibodies in all tested serum samples targeted glycosylated epitopes, ranging from 13% to 88% in the 16 samples we analyzed. In contrast, the anti-YghJ IgA in lavage mainly targeted non-glycosylated epitopes, with only 4 of 11 volunteers having a substantial proportion targeting glycosylated epitopes.

This finding was surprising given that we expected the antigen-specificity of systemic IgA in a volunteer to be similar to that of the volunteer’s gut mucosal IgA. A potential explanation might be that, in some volunteers, intestinal IgA targeting glycosylated epitopes is cross-reacting with other glycosylated microbial target proteins, thus depleting them so they appear lower in intestinal lavage samples. Another possibility is that the gut anti-YghJ IgA response is actively limiting its response towards the potentially more strain-specific glycosylated epitopes in some volunteers. We are not aware of any other studies that have investigated differential systemic and intestinal antibody specificities against glycosylated epitopes, but our finding parallels the findings that the proportion of anti-citrullinated protein IgA antibodies is lower in saliva than in serum in patients with rheumatoid arthritis (30).

To be able to study the IgA antibody response against glycosylated epitopes on YghJ, we produced native YghJ by expressing its gene under its native promoter in the wild-type TW10722 strain to ensure that the native level of glycosylation was maintained. The results from our BEMAP analyses suggest that the YghJ we produced was heavily glycosylated, as expected. In a recently completed study (31), BEMAP analyses on YghJ isolated in a similar manner from ETEC reference strain H10407 showed that this YghJ also was O-linked hyper-glycosylatedThe BEMAP analyses only identifies amino acid residues that are glycosylated to some degree, but it does not provide a measure of what proportion of proteins are actually glycosylated at a given residue. We expect that YghJ expressed by TW10722 is normally not fully glycosylated at all identified residues given there was no discernable size difference between the glycosylated and non-glycosylated variants when analyzed by SDS-PAGE (**Figure 1**). The lack of a clear size difference was not unexpected since this has also been observed for several other large *E. coli* glycoprotein virulence factors, including the TibA (31), EtpA (32), and the AIDA-I (33).

Some volunteers appeared to have high pre-existing levels of anti-YghJ IgA antibodies both in serum and in lavage, with day 0 levels being 5- and 11-fold higher in serum and lavage, respectively, than the median levels. Interestingly, these volunteers also exhibited some of the strongest anti-YghJ antibody responses from the TW10722 infection. Some degree of pre-existing immunity against YghJ may not be uncommon since both pathogenic and many commensal *E. coli* often produce YghJ (7).

Given the nature of the lavage sample collection, where volunteers need to drink large quantities of a laxative, variation in antibody levels between different lavage samples is expected. The variation is caused by differences in the amount of liquid consumed, differences in intestinal peristalsis, or the presence of contaminants that increase background or neutralize antibodies. To compensate for this variation, we estimated the anti-YghJ IgA levels by using arbitrary units instead of MFI values, and we normalized the estimates by adjusting for the total IgA concentration in the lavage samples. Gut inflammation associated with infection may prompt bystander activation of B cells in GALT and increased production of polyclonal IgA antibodies (34). Although not statistically significant, the median total IgA concentration found in the lavage samples had increased 2-fold from day 0 to day 10. Normalizing against total IgA, therefore, has likely resulted in a conservative estimate of YghJ-specific fold changes.

In conclusion, we found that infection with ETEC strain TW10722 induced strong systemic and mucosal anti-YghJ IgA antibody responses and that substantial proportions of serum IgA antibodies target glycosylated YghJ epitopes. However, in most volunteers, only a small proportion of anti-YghJ IgA found in gut lavage seemed to target glycosylated epitopes. These findings warrant further investigation into how careful application of vaccine antigen glycosylation could help to make YghJ-based vaccines more immunogenic, and potentially more broadly protective against a wide range of pathogenic *E. coli*.

## Data Availability Statement

All data are given in the article and supplementary table. The TW10722 BEMAP data have been deposited to the ProteomeXchange Consortium via the PRIDE (35) partner repository with the dataset identifier PXD030322.

## Ethics Statement

The studies involving human participants were reviewed and approved by REC-West (case number 2014/826). The participants provided their written informed consent to participate in this study.

## Author Contributions

SR, HS, AA, AB, and KH conceived and designed the experiments. SR, HS, AB, and KH wrote the first draft of the manuscript. SR, HS, AA, AB, and KH analyzed data. SR and KH performed the bead assay experiments. MT performed cloning and generated strains used for producing YghJ. AB produced the YghJ and analyzed the mass spectrometry data. All authors contributed to the manuscript and approved the submitted version.

## Funding

This work was supported by the University of Bergen, The Research Council of Norway (project number 234364), and Innovation Fund Denmark (Grant # 7041-00220).

## Conflict of Interest

AB is listed as an inventor on a patent relating to WO 2017/059864 held by the University of Southern Denmark and Aarhus University. AB and AA have a financial interest in GlyProVac ApS, which has licensed exclusively the IP stated above. AB is the scientific founder, shareholder, and a member of the board. AA is a co-founder, shareholder, and a member of the board. AB, AA, and MT are employees of GlyProVac ApS.

The remaining authors declare that the research was conducted in the absence of any commercial or financial relationships that could be construed as a potential conflict of interest.

## Publisher’s Note

All claims expressed in this article are solely those of the authors and do not necessarily represent those of their affiliated organizations, or those of the publisher, the editors and the reviewers. Any product that may be evaluated in this article, or claim that may be made by its manufacturer, is not guaranteed or endorsed by the publisher.
