# Supplementary material for: Characterization of Glycosylation-Specific Systemic and Mucosal IgA Antibody Responses to Escherichia coli Mucinase YghJ (SslE)
Source: Front Immunol. 2021 Dec 17;12:760135. doi: 10.3389/fimmu.2021.760135 (PMC8718676; doi:10.3389/fimmu.2021.760135)

Supplementary information

Riaz S et al. Characterization of glycosylation-specific systemic and mucosal IgA antibody responses to *Escherichia coli* mucinase YghJ (SslE)

Supplementary table S1: Strains, plasmids and cell lines used in this study.

**Strains**	**Genotype**	**Antibiotic resistance**	**Reference**
ETEC TW10722			(1)
E. coli Top10	F- *mcrA* Δ(*mrr-hsd*RMS-*mcrBC*) ϕ80*lac*ZΔM15 Δ*lac*X74 *nup*G *rec*A1 *ara*D139 Δ(*ara-leu*)7697 *gal*E15 *gal*K16 *rps*L(StrR) *end*A1 λ-. Used for cloning		Invitrogen
MG1655Δ*hldE*	*hldE* isogenic knockout strain		(2)
TW10722*yghJ*	3xFLAG epitope tagging of yghJ on chromosome		This study
**Plasmids**			
pKD46	Red recombinase expression vector. Temperature sensitive – replicates at 30C	Amp^R	(3)
pSUB11	Used for 3xFLAG epitope tagging on chromosome	Kan^R	(4)
pXG-0	Low copy number plasmid	Cml^R	(5)
pGPV106	Plasmid used for overexpression of ETEC TW10722 *yghJ* 3xFLAG	Cml^R	This study

References to supplementary table S1.

1. Sakkestad ST, Steinsland H, Skrede S, Lillebo K, Skutlaberg DH, Guttormsen AB, et al. A new human challenge model for testing heat-stable toxin-based vaccine candidates for enterotoxigenic Escherichia coli diarrhea - dose optimization, clinical outcomes, and CD4+ T cell responses. PLoS Negl Trop Dis. 2019;13(10):e0007823.
2. Maigaard Hermansen GM, Boysen A, Krogh TJ, Nawrocki A, Jelsbak L, Moller-Jensen J. HldE Is Important for Virulence Phenotypes in Enterotoxigenic Escherichia coli. Front Cell Infect Microbiol. 2018;8:253.
3. Datsenko KA, Wanner BL. One-step inactivation of chromosomal genes in Escherichia coli K-12 using PCR products. Proceedings of the National Academy of Sciences of the United States of America. 2000;97(12):6640-5.
4. Uzzau S, Figueroa-Bossi N, Rubino S, Bossi L. Epitope tagging of chromosomal genes in Salmonella. Proc Natl Acad Sci U S A. 2001;98(26):15264-9.
5. Urban JH, Vogel J. Translational control and target recognition by Escherichia coli small RNAs in vivo. Nucleic Acids Res. 2007;35(3):1018-37.

Supplementary table S2: Non-adjusted anti-IgA Median Fluorescence Intensity (MFI) values for non-glycosylated and glycosylated YghJ (nYghJ and gYghJ) at day 0 and day 10 before convertion into arbitrary units (AUs).

	Lavage anti-IgA levels in MFI						Serum anti-IgA levels in MFI					
	anti-nYghJ IgA			anti-gYghJ IgA			anti-nYghJ IgA			anti-gYghJ IgA		
ID	day 0	day10	ratio d10/d0	day 0	day10	ratio d10/d0	day 0	day10	ratio d10/d0	day 0	day10	ratio d10/d0
EV01	209	1418	6.78	135	974	7.21	252	685	2.72	314	1702	5.42
EV02	1462	2095	1.43	1141	6631	5.81	454	694	1.53	694	3983	5.74
EV03	2599	2686	1.03	1004	2549	2.54	413	630	1.53	568	830	1.46
EV04	646	1579	2.44	666	3739	5.61	639	1051	1.64	1618	6349	3.92
EV05	3539	31114	8.79	1633	16691	10.22	362	2670	7.38	717	6777	9.45
EV06	547	18676	34.14	416	14618	35.14	535	4721	8.82	750	6588	8.78
EV07	329	322	0.98	378	1828	4.84	538	475	0.88	945	3022	3.20
EV08	899	4771	5.31	871	5060	5.81	638	1145	1.79	895	3145	3.51
EV09	258	27607	107.0	266	28342	106.5	1101	3052	2.77	1576	6107	3.88
EV19	1532	336	0.22	158	1161	7.35	650	988	1.52	946	3448	3.64
EV21	173	345	1.99	111	438	3.95	369	708	1.92	608	2368	3.89
EV22	207	610	2.95	152	593	3.90	420	879	2.09	718	2957	4.12
EV23	181	3554	19.64	177	6836	38.62	391	1270	3.25	599	3918	6.54
EV24	na	na	na	na	na	na	1089	3547	3.26	1509	5451	3.61
EV25	1529	6028	3.94	758	3413	4.50	403	1295	3.21	647	3083	4.77
EV26	204	305	1.50	155	765	4.94	590	848	1.44	1512	3266	2.16
EV27	142	521	3.67	68	880	12.94	574	892	1.55	1204	4193	3.48
EV28	186	1689	9.08	135	1558	11.54	487	884	1.82	774	2466	3.19
EV29	550	3723	6.77	477	3325	6.97	774	1489	1.92	1424	4924	3.46
EV30	574	1251	2.18	438	660	1.51	1335	3800	2.85	1913	9817	5.13
EV31	na	na	na	na	na	na	1538	2447	1.59	1636	3386	2.07

na: not applicable due to very low IgA amount in lavage sample

Supplementary table S3: Total lavage IgA (mg/mL) data used to normalize anti-YghJ lavage IgA levels.

Lavage total IgA levels		
ID	day 0	day 10
EV01	0.56	0.73
EV02	0.63	0.83
EV03	0.73	1.42
EV04	3.32	0.94
EV05	0.60	1.08
EV06	0.36	2.56
EV07	1.17	1.15
EV08	0.46	1.53
EV09	0.74	1.53
EV19	1.06	0.12
EV21	0.58	0.68
EV22	0.18	0.13
EV23	2.04	1.57
EV24	<0,02	<0,02
EV25	3.48	2.23
EV26	0.61	0.68
EV27	1.37	2.52
EV28	0.88	1.55
EV29	2.76	2.16
EV30	3.33	2.77
EV31	<0,02	6.60
Median	0.74	1.47

Supplementary table S4: Serum anti-YghJ IgA levels (in arbitrary units), with and without depletion of IgG and IgM. Depletion of IgG and IgM resulted in significantly increased IgA responses and comparability of pre-incubated samples included in the specificity assay.

	Serum anti-YghJ IgA levels **without** IgG and IgM depletion		Serum anti-YghJ IgA levels **with** IgG and IgM depletion	
	anti-nYghJ IgA	anti-gYghJ IgA	anti-nYghJ IgA	anti-gYghJ IgA
**EV01**	236	324	256	168
**EV02**	178	2254	64	2522
**EV04**	329	1395	1103	2966
**EV05**	1464	3119	7903	8317
**EV06**	4718	4117	7878	7871
**EV07**	104	961	98	527
**EV08**	626	2440	1095	2219
**EV09**	1602	1853	4586	6140
**EV19**	226	450	1269	1317
**EV23**	514	909	1114	2129
**EV24**	2072	1590	5898	3276
**EV27**	441	647	495	1195
**EV28**	309	312	244	645
**EV29**	774	918	1506	2314
**EV30**	3131	4749	7895	10928
**EV31**	1881	1237	3809	1096
**Mean**	1163	1705	2826	3352
**Median**	570	1316	1192	2266

Supplementary table S5: Pilot data showing optimization of protein required for depletion of specific YghJ antibodies in specificity assay (values given in median fluorescence intensity).

**Pre-incubation protein and amount**	**nYghJ protein**					
	**1 µg**	**0.5 µg**	**0.25 µg**	**1 µg**	**0.5 µg**	**0.25 µg**
**Antibody levels**	**anti-nYghJ IgA level**			**anti-gYghJ IgA level**		
**EV06 day 10**	552	880	869	1004	1295	1206
**EV09 day 10**	1127	1121	1718	6363	5983	7111

**Pre-incubation protein and amount**	**gYghJ protein**					
	**1 µg**	**0.5 µg**	**0.25 µg**	**1 µg**	**0.5 µg**	**0.25 µg**
**Antibody levels**	**anti-nYghJ IgA level**			**anti-gYghJ IgA level**		
**EV06 day 10**	556	796	14516	352	550	12296
**EV09 day 10**	849	1121	28478	1860	2946	27806

**Pre-incubation protein and amount**	**Both nYghJ and gYghJ proteins**					
	**1 µg**	**0.5 µg**	**0.25 µg**	**1 µg**	**0.5 µg**	**0.25 µg**
**Antibody levels**	**anti-nYghJ IgA level**			**anti-gYghJ IgA level**		
**EV06 day 10**	608	507	540	528	488	535
**EV09 day 10**	634	864	1159	1345	2734	4228

Supplementary table S6: BEMAP analysis of glycosylated TW10722 YghJ. YghJ was digested with Trypsin and O-linked glycosylated peptide sequences were identified using BEMAP in combination with mass spectrometry. The start and end position of each of the 27 peptide sequences within YghJ are listed, as well as the specific residue number which is modified. Lower case s or t indicate modified residue.

Start	End	Sequence	Site#	Site#	Site#	Site#	Site#	Site#	Site#	Site#	Site#
91	101	TGYLTLGGsLR	S99								
102	145	VtGDItCNDEssDGFtFtPGDKVtCVA GNNttIATFDTQSEAAR	T103	T107	S112	S113	T117	T119	T125	T132	T133
153	170	VsFsLEDAQELAGsDNKK	S154	S156	S166						
281	300	LVDsQGDGVVGVNYYtNsGR	S284	T296	S298						
438	444	WFsLTTR	S440								
458	466	LWGVDtNYK	T463								
471	488	FHVFHDstNFYGstGNAR	S477	T478	S483	T484					
489	506	GQAVVNIsNAAFPILMAR	S496								
544	562	ETATFNLPFIsLGQVGDGK	S554								
577	611	CPNGYsWNGGVNKDGQCTLNSDPD DmKNFmENVLR	S582								
590	611	DGQCtLNsDPDDMKNFMENVLR	T594	S597							
612	617	YLsNDR	S614								
624	638	ssMTVGTNLDTVYFK	S624	S625							
640	654	HGQVLGnsAPFAFHK	S647								
722	745	GGsVLIMENVMsNLKEEsASGFVR	S724	S733	S739						
746	758	LLDAAGLsMALnK	S753								
759	773	sVVNNDPQGYPDRVR	S759								
808	832	YQQENKPDDKPKLEVAsWQEEVEGK	S824								
847	855	TPEsLAAAK	S850								
1079	1095	WFAGNMQsTGLWAPAQK	S1086								
1101	1122	sNANVPVTVTVALADDLTGREK	S1101								
1136	1146	TYsLDASGTVK	S1138								
1159	1176	GDsKDNEsAsFTFTGVVK	S1161	S1166	S1168						
1182	1209	DGAWKNDLnsPAPLGELESDAFVYTAPK	S1191								
1210	1222	KNLNAsNYTGGLK	S1215								
1223	1249	QFANDLDtFAssMNDFYGRNEEDGKHR	T1230	S1233	S1234						
1343	1364	VADDITVAPEYLEEsNGqAWAR	S1357								

Supplementary figure 1. Correlation between lavage and serum fold changes for anti-nYghJ and anti-gYghJ antibodies.

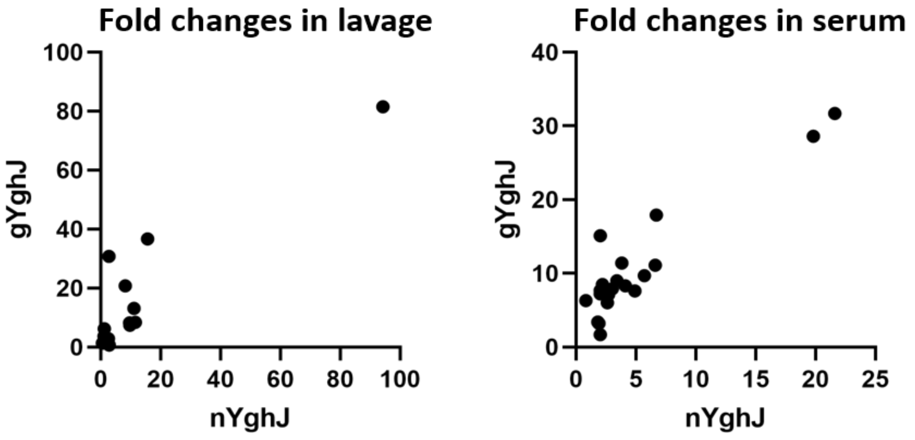

Supplementary figure 2. Correlation between nYghJ and gYghJ fold changes in lavage and in serum.

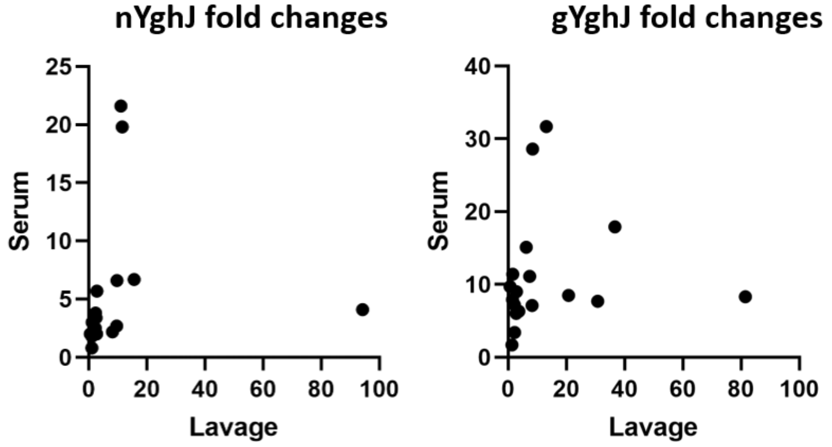

Supplement: Supplementary file 1 [file DataSheet_1.pdf]
